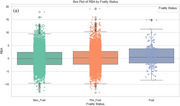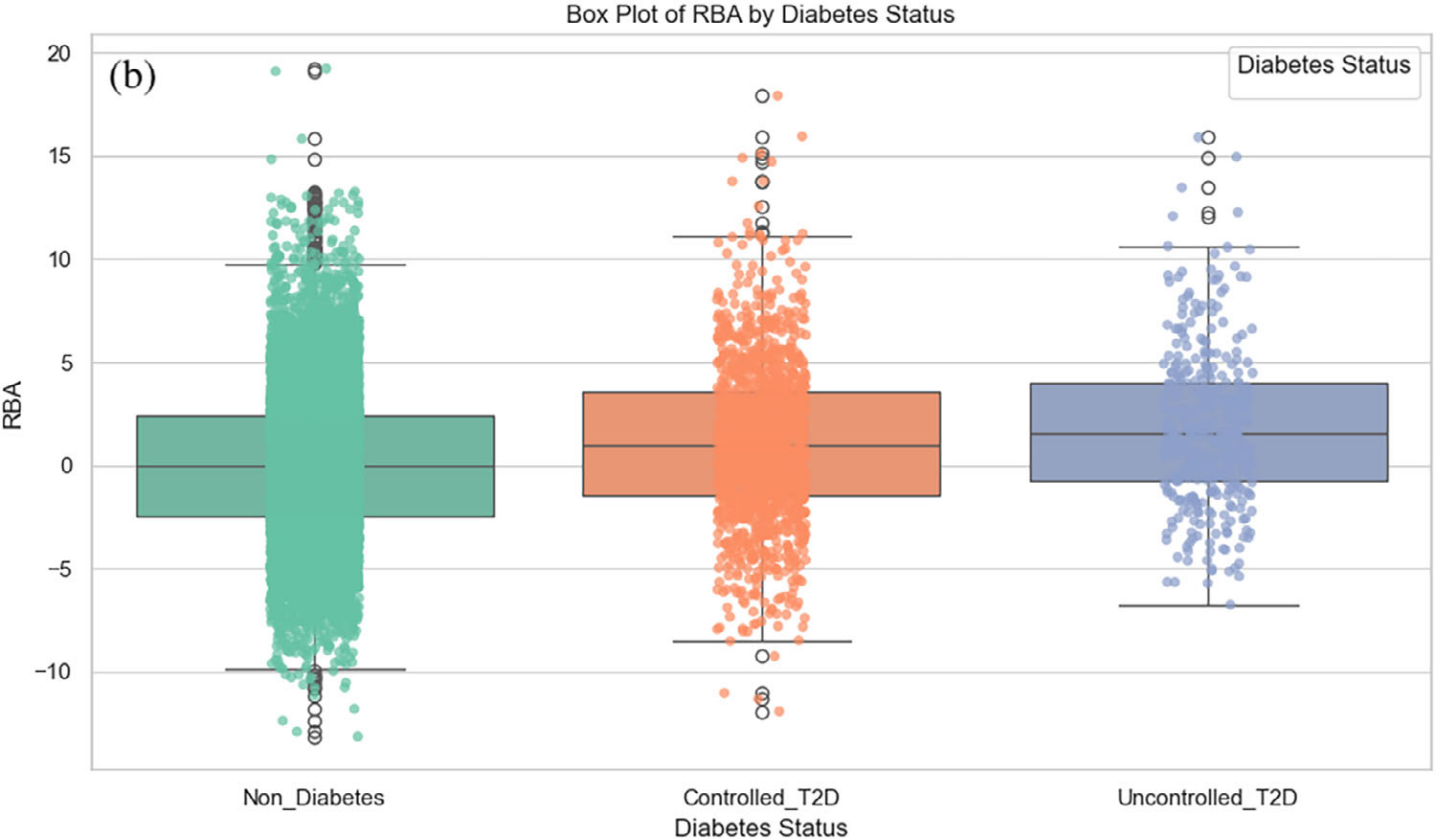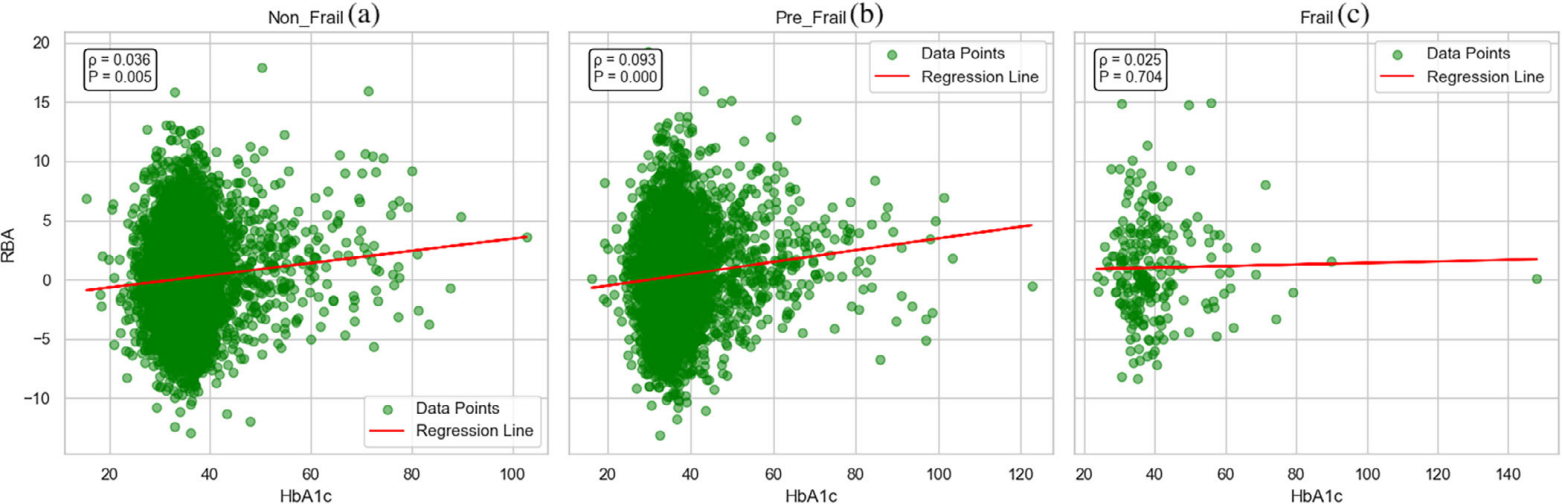# Investigating the Association of Frailty Score and Diabetes with Relative Brain Age : Insights from the UK Biobank

**DOI:** 10.1002/alz70856_103010

**Published:** 2025-12-26

**Authors:** Mahboubeh Motaghi, Olivier Potvin, Iman Beheshti, Simon Duchesne

**Affiliations:** ^1^ Laval University, Quebec City, QC, Canada; ^2^ Quebec Heart and Lung Institute, Quebec city, QC, Canada; ^3^ Quebec Heart and Lung Institute, Québec, QC, Canada; ^4^ University of Manitoba, Winnipeg, MB, Canada; ^5^ Kleysen Institute for Advanced Medicine, Health Science Centre, Winnipeg, MB, Canada; ^6^ Quebec Heart and Lung Institute Research Centre, Quebec City, QC, Canada

## Abstract

**Background:**

Aging and type 2 diabetes (T2D) are both associated with brain cortical atrophy and structural brain changes. Frailty also appears linked to cortical atrophy and reduced brain volume. The joint impact of T2D and frailty on brain health is not known. This study investigates the association between frailty and T2D with relative brain age (RBA), a cortical thickness‐based measure reflecting deviations from normative brain aging trajectories, in the UK Biobank.

**Method:**

We selected UK Biobank participants aged ≥55 years for whom an MRI was available. T2D status was classified as non‐diabetic, controlled (HbA1c 6.5–7%), or uncontrolled (HbA1c >7%). Frailty was assessed using an adapted Cardiovascular Health Study phenotype, categorizing participants as non‐frail, pre‐frail, or frail. RBA was estimated from cortical thickness and regional brain volume data using the Brain AGE framework, which includes age‐bias correction. Linear regression examined the relative contributions of age, sex, HbA1c, and frailty, and their interaction (HbA1c_Frailty_Interaction) to RBA. ANCOVA and post‐hoc analyses assessed group differences, and box plots illustrated RBA by frailty (Figure 1a) and diabetes status (Figure 1b), while scatterplots visualized the interaction effects (Figure 2).

**Result:**

As expected, age showed no significant association (*p* = 0.29) with bias‐corrected RBA. Being male was associated with higher RBA (β=1.023, *p* <0.001). Linear regression identified HbA1c and frailty as significant predictors of RBA. Higher HbA1c levels and frailty scores were associated with elevated RBA, explaining 28.1% and 5.2% of variance, respectively. ANCOVA revealed significant effects of HbA1c (F=57.79, *p* <0.001) and frailty (F=6.46, *p* = 0.0016) on RBA. Post‐hoc analysis showed that frail participants had significantly higher RBA compared to non‐frail and pre‐frail groups (Figure 1a). Uncontrolled diabetes was associated with the highest RBA, exceeding both controlled diabetes and non‐diabetic groups (Figure 1b). Furthermore, a significant interaction was observed between HbA1c and frailty (β=0.007, *p* <0.001), indicating that HbA1c had a deleterious effect on RBA, but not individuals with frailty (Figure 2).

**Conclusion:**

Frailty and poor glycemic control (HbA1c >7%) independently contribute to accelerated brain aging, as indicated by elevated RBA and their effects are modified according to one and other. Targeted strategies addressing these factors are crucial for reducing brain aging and dementia risk.